# Health Symptoms Associated with Pesticides Exposure among Flower and Onion Pesticide Applicators in Arusha Region

**DOI:** 10.29024/aogh.2303

**Published:** 2018-08-31

**Authors:** Suten Geofrey Mwabulambo, Ezra Jonathan Mrema, Aiwerasia Vera Ngowi, Simon Mamuya

**Affiliations:** 1 Department of Environmental and Occupational Health, School of Public Health and Social Sciences, Muhimbili University of Health and Allied Sciences, Dar es Salaam, TZ

## Abstract

**Introduction:**

Pesticides are extensively used in agriculture to control harmful pests and prevent crop yield losses or product damage. In Tanzania several studies have been conducted on health effects of pesticides on agricultural workers. However, there are few studies on neurological health symptoms associated with pesticide exposure in flower and onion farms.

**Objective:**

The objective of this cross-sectional study was to assess health symptoms associated with pesticide exposure among flower and onion pesticide applicators in the Arusha region, Tanzania.

**Methods:**

Data on demographic variables and health symptoms associated with pesticide exposure were collected from 140 males who were employed in spraying pesticides on flower and onion farms in Arusha, Meru and Karatu districts between April and May 2017. The study participants were interviewed using a structured questionnaire with questions focusing sociodemographic characteristics, occupation, pesticide exposure, common type of pesticide used in the area and neurological symptoms experienced during and after pesticide spraying. To determine the intensity of pesticide exposure, acetylcholinesterase assay was done by using the Test-mate Model 400 device with a photometric sensor.

**Results:**

Ninety-five percent of pesticide applicators reported handling organophosphate pesticides. Body weakness was the most frequently reported neurological symptom (57.1%) followed by perspiration and headache (40.7%), poor appetite and depression (29.3%) and irritation (26.4%). About 27% of pesticide applicators had an acetylcholinesterase level below the limit value.

**Conclusion:**

A high proportion of neurological health symptoms and cholinesterase test depression was noted among pesticide applicators in both farms. There is a need to conduct further studies to ascertain causality for such high instances of neurological symptoms.

## Background

Globally, approximately five billion pounds of pesticides are used per annum, of which organophosphate and carbamate insecticides, dithiocarbamate fungicides and phenoxyl herbicides are the most commonly used [1]. Developing countries are known to use less than 20% of the world production of agrochemicals including pesticides. Africa consumes about 4% of the total pesticides produced in the world [2]. About 81% of pesticides used in Tanzania are in the agricultural sectors [3].

Due to globalization and local population growth, demand for food and raw materials in Tanzania led to a shift to commercial agriculture in the 1980s [4], which required the use of hazardous chemicals like synthetic fertilizers and pesticides [4]. Arusha Region is known to be the leader in pesticide trading and utilization because of the intensification of horticulture. Intensive cultivation of horticultural crops (vegetables, fruits, flowers, spices) in Tanzania has increased significantly to meet both local and international market demand. Farming is carried out both by large- and small-scale farmers. Pesticide applicators are mostly young men who work as casual laborers, sometimes with no knowledge or training about the environmental and health effects of pesticides and are thus ill-equipped to deal with exposures and their effects. Uses of protective measures during pesticide application depend on availability, affordability, comfortability and regulations, and thus are limited in small-scale farming areas. In addition, without proper protection, pesticide exposure risk and the occurrence of adverse health effects increase.

About 99% cases of pesticide poisoning fatalities occur only in developing countries, although these countries consume 20–25% of pesticides [5]. Pesticide applicators face great risks of exposure to toxic pesticides that were banned or restricted due to incorrect application techniques, poorly maintained or totally inappropriate spraying equipment, inadequate storage practices, and often reuse of old pesticide containers for food and water storage [6]. Several adverse health effects, such as temporary acute effects like irritation of eyes and excessive salivation may result from exposure to pesticides. Effects on the central nervous system (CNS) like restlessness, loss of memory, convulsions and coma are also common. Effects on the parasympathetic and sympathetic nervous system, such as respiratory paralysis, have been widely reported [7].

Occupational health and safety and workers’ compensation laws in Tanzania cover only formal economic sectors. Large-scale horticulture, particularly flower farms, is bound by the laws to ensure health and safety of their workers. The international horticultural market under fair trade also controls the use of pesticides. Most small-scale horticultural farm activities are informal and thus do not receive occupational health services in the country but rely on primary health care. Understanding the differences in pesticide exposures and health effects among pesticide applicators in large- and small-scale farms is essential in guiding resource allocation, informing policy and control efforts.

Pesticide uses in agriculture activities expose applicators to risk factors for neurological health symptoms. Little has been done in Tanzania on neurological health symptoms and the risk factors associated with pesticide exposure to applicators. The present study was initiated to assess the neurological health symptoms and associated risk factors among the pesticide applicators. Specifically, we identified the pesticides used in flowers and onions farms. The intensity of pesticide exposure among applicators in selected farms was measured by AChE assay, self-reported neurological symptoms related to pesticide exposure and observation of personal protective measures employed during pesticide application. The study conducted interviews for symptoms related to pesticide exposure and measured whole blood cholinesterase activity to determine intensity of exposure. It also observed use of personal protective equipment.

## Materials and Methods

A cross-sectional study was conducted by collecting demographic and health symptom data from 140 male pesticide applicators in flower and onion farms in Arusha, Meru and Karatu districts between April and May 2017 in the Arusha region. Multistage random sampling was used to select the study subjects from the lists of 216 contractual and 182 casual workers. The lottery method was used to select three districts among the seven in Arusha region. Two wards from each of the participating districts were randomly selected: Olorien and Olmotonyi of Arusha district, Moivaro and Mlangarini of Meru district and Barai and Mang’ola Chini of Karatu district. One village from each ward was selected, randomly generating a total of six participating villages. The systematic random sampling was used to select seven farms from six villages. Lists of pesticide applicators were solicited from farm managers and a total of 20 pesticide applicators were selected randomly from seven selected farms, generating a total of 140 study participants.

The study participants were interviewed using a structured questionnaire developed by biological monitoring of pesticide exposures [8] and adapted to the local situation including translation into Swahili language. Prior to data collection, the questionnaire was pretested among 10 pesticide applicators working in vegetables gardens in Kariakoo ward, Dar es Salaam. After pretesting, ambiguous questions were identified and adjusted accordingly. The duration for administering the questionnaire was determined.

Data collection was done by the principal investigator of the study. The investigator visited the pesticide applicators in the onion and flower farms and collected data by using a pretested and structured questionnaire via face-to-face interviews with the applicators. The questions focused on sociodemographic characteristics, occupation, pesticide exposure, common type of pesticide used in the area, the use of personal PPE and neurological symptoms experienced during and after pesticide spraying. The Q16 questionnaire contains 16 modified questions, which were used for evaluation of neurological health symptoms that were answered by either “yes” or “no” [9,10].

The observation checklist was used to collect information on how PPE were worn; quality of PPE; hazards from pesticide spills; pesticide preparation process before spraying; storage and disposal of pesticide empty containers; and personal hygiene after spraying. Visual media collection using camera was done to collect information on PPE’s uses, pesticide handling and spraying process.

Erythrocyte acetylcholinesterase level, which is indicative of exposure to cholinesterase inhibiting organophosphate and carbamate insecticides, was determined from all 140 participants. The medical laboratory scientist from Tropical Pesticide Research Institute in Tanzania carried out the blood test for AChE activities on the same day of the interview. In brief, fingers were washed with alcohol and dried for few seconds. Ten microliters of capillary blood was drawn using a finger prick sterile lancing device and placed into the assay tube. AChE erythrocyte cholinesterase reagent was then dissolved in distilled water and inserted into the analyzer. The analyzer provided the measurements of haemoglobin, the level of AChE and haemoglobin adjusted erythrocyte acetylcholinesterase activity. The AChE kit used for AChE assay is approved for field use by the World Health Organization (WHO). The WHO limit was used to identify the levels that were lower and above the limit on the cholinesterase test to pesticide applicators in onion and flower farms [11].

A paired t test was used to compare the mean of AChE levels between pesticide applicators of onion and flower farms. The test was also used to compare the number of neurological symptoms reported between the two groups.

## Results

### Demographic characteristics of study participants

The demographic characteristics of the pesticide applicators in flower and onions farms are presented in Table 1. The study participants were all males whose mean age is 30.63 ± 6.048 and 28.02 ± 7.557 years for flower and onion farms, respectively. A majority of pesticide applicators in onion farms (62.5%) were below 30 years of age and 5.4% were below 18 years. Most were casual workers (94.6%) with one third having work experience of less than a year with no training on pesticide safety or management. Almost half of the workers reported working more than eight hours per day. In flower farms 52.4% of pesticide applicators were aged between 29 and 38 years and most (95.2%) worked on a contract basis. A majority (77%) reported working between one and five hours per day.

**Table 1:** General characteristics of the study participants in flower farms (n = 84) and onion farms (n = 56) in Arusha Region.

Characteristics	Flower farms (%)	Onion farms (%)

Age (in years)		

18 and below	0	5.4
19–28	36.9	57.1
29–38	52.4	26.8
39 and above	10.7	10.7
**Level of education**		

No formal education	7.1	5.4
Primary education	78.6	83.9
Secondary education	14.3	10.7
**Employment**		

On contract	95.2	1.8
Casual	4.8	94.6
Self-employed	0	3.6
**Attended training on pesticide**		

Yes	86.9	0
No	13.1	100
**Work experience (in years)**		

<1	3.6	33.9
1–5	77.4	50.1
6–10	16.7	7.1
>11	2.4	8.9
**Work duration (hours per day)**		

<8	100	42.9
>8	0	57.1

### The relationship between demographic characteristics and AChE level

The result shows those pesticide applicators who are 19–28 years of age were experience AChE test below range in both groups. More than 50% of pesticide applicators that had a primary level of education had AChE below normal range in onion and flower farms, and a high proportion of those who worked one to five years had AChE below the normal range. All demographic factors was not statistically significant as compared to AChE test results (p > 0.005) (Table 2).

**Table 2:** Relationship between Demographic factors and AChE Test.

Demographic factors	AChE test limit in onion (N = 56)			AChE test limit in flower (N = 84)		

	AChE within normal range	AChE below normal range	P Value	AChE within normal range	AChE below normal range	P Value

Age group						

<18	1(33.3%)	2(66.7%)		0(0.0%)	0(0.0%)	
19–28	19(59.4%)	13(40.6%)	0.795	24(77.4%)	7(22.6%)	
29–38	10(66.7%)	5(33.3%)		36(81.2%)	8(18.2%)	0.785
>39	4(66.7%)	2(33.3%)		8(88.9%)	1(11.1%)	
**Education Level**						

No formal Education	2(66.7%)	1(33.3%)		5(83.3%)	1(16.7%)	
Primary Education	29(61.7%)	18(38.3%)	0.854	55(83.1%)	11(16.7%)	0.294
Secondary Education	3(50.0%)	3(50.0%)		8(66.7%)	4(33.3%)	
**Work experience (in years)**						

<1	11(57.9%)	8(42.1%)		3 (100%)	0(0.0%)	
1–5	17(60.7%)	11(39.3%)	1.000	51(78.5%)	14(21.5%)	
6–10	3(75.0%)	1(25.0%)		13(92.9%)	1(7.1%)	0.276
>11	3(60.0%)	2(40.0%)		1(50.0%)	1(50.0%)	

### Common pesticides used for crops protection

Organophosphates were the most common type of pesticides used, accounting for 98%, whereas carbamates accounted for only 2% (Tables 3 and 4). In flower farms, 75% of pesticides belonged to the organochlorine group, 25% were pyrethroid. Eighty-one percent of pesticides used in onion farms belong to organochlorine and 19% were pyrethroids, which were used for pest control. The result shows that 54.1% of pesticides were moderately hazardous (II), 21.6% slightly hazardous (III) and 24.3% were unlikely to represent any acute hazard (IV) in normal use according to WHO criteria.

**Table 3:** The list of pesticides used in flowers fields.

Trade name of pesticide	Name of the active substance(s)	Class	Type of Pesticide	Approved uses	Reference on health effects

Amsac	Indoxacarb 14.50%	II	Insecticide	For the control of African boll worm on vegetable	US EPA
Merpan	Captan 50%	III	Fungicide	For the control of powdery mildew on flowers.	Strivastava and Kesavachandran
Golan	Acetamiprid 50%	II	Insecticide	For the control of aphids, thrips, leaf minor & flea beetle on flowers	US EPA
Ippon	Iprodione 50%	III	Fungicide	For the control of botrytis on flowers.	US EPA
Impulse	Spiroxamine 50%	II	Fungicide	For the control of powdery mildew on flowers.	US EPA
Ortiva	Azoxystrobin 35%	IV	Fungicide	For the control of rust, botrytis & downy mildew on flowers.	Strivastava and Kesavachandran
Previcur energy	Fosetyl-aluminium + propamocarb 310 g/l	III	Fungicide	For the control of botrytis, pythium & phytophthora on flowers.	Strivastava and Kesavachandran
Previcur N	Propamocarb hydrochloride 722 g/l	III	Fungicide	For the control of downy mildew on flowers.	Strivastava and Kesavachandran
Proplant SL	Propamocarb hydrochloride 722 g/l	III	Fungicide	For the control of downy mildew on flowers.	Strivastava and Kesavachandran
Ridomil Gold	Mancozeb 64%+ metalaxil 4%	IV	Fungicide	For the control of downy mildew on grape.	Strivastava and Kesavachandran
Tracer 480 SC	Spinosad 480%	III	Insecticide	For the control of thrips and leaf miners on flowers	US EPA
Round-up	Glyphosate 420 g/l	IV	Herbicide	For the control of annual & perennial weeds	
Teldor	Fenhexamid 50%	III	Fungicide	For the control of Botrytis on flowers.	Strivastava and Kesavachandran
Scala SC	Pyrimethanil 400 g/l	IV	Fungicide	For the control of Botrytis on flowers.	Strivastava and Kesavachandran
Ridomil MZ	Metalaxyl 5%	III	Fungicide	For the control of downy mildew on grape.	Strivastava and Kesavachandran
Collis	Kresoxim-methyl + boscalid 100 g/l	II	Fungicide	For the control of powdery mildew on flowers.	Strivastava and Kesavachandran
Nimrod 25EC	Bupirimate 250 g/l	III	Fungicide	For the control of powdery mildew on pepper.	Strivastava and Kesavachandran
Poller/infinito	Propamocarb hydrochloride 722 g/l	III	Fungicide	For the control of powdery mildew on flowers.	Strivastava and Kesavachandran
Polar	Polyoxin 50%	IV	Fungicide	For the control of powdery mildew on strawberry	Strivastava and Kesavachandran
Proven 1,9EC	Emamectin + benzoate 1.90%	III	Insecticide	For the control of stalk borer	US EPA

Key reference: US EPA: United State Environmental Protection Agency, (Reregistration eligibility decision for triforine) [12,13,14].

**Table 4:** The list of pesticides used in onion fields.

Trade name of pesticide	Active ingredient	Class	Type of Pesticide	Approved uses	Reference on health effects

Supercron 500EC	Profenofos 500 g/lEC	II	Insecticide	For the control of aphids (Acyrtosiphon pisum)	US EPA
Snowcron 500EC	Profenofos 500 g/lEC	II	Insecticide	For the control of aphids (Acyrtosiphon pisum)	US EPA
Wilcron profenos 500EC	Profenofos 500 g/lEC	II	Insecticide	For the control of fruit worm	US EPA
Bicron 500EC	Profenofos 500 g/lEC	II	Insecticide	For the control of onion thrips	US EPA
Select Plus	Profenofos 300 g/lt + lambdacylothrine 5 g/lt	II	Insecticide	For the control of onion thrips (thrips tabaci in onion)	US EPA
Dudumectin	Emamectin 4.8% + acetameprid 6.4%	II	Insecticide	For the control of green aphids	US EPA
Protrin 60EC	Chloropyrifos 500 g/lt + cypermethrin 100 g/lt	II	Insecticide	For the control of tuber moth (PTM) and African boll worm (ABW)	US EPA
Prosper 720EC	Profenofos 600 g/lt + cypermethrin 120 g/lt	III	Insecticide	For the control of onion thrips on onion.	US EPA
Snowsoldier 240EC	Carbonsulfan 240 g/lt	II	Insecticide	For the control of locust and grasshoppers.	US EPA
Marshal 240EC	Carbosulfan	II	Insecticide	For the control of locust and grasshoppers.	US EPA
Lamdex 5EC	Lambda-cyhalothrin 5% EC	II	Insecticide	For the control of stalk	US EPA
Ninja plus 5EC	Lambda cylothrin (pyrethroid) 50 g/lt	II	Insecticide	For the control of stalk	US EPA
Sumo 5EC	Lambda cylothrin (pyrethroid) 50 g/lt	II	Insecticide	For the control of stalk	US EPA
Trigger 5EC	Lambda cylothrin (pyrethroid) 50 g/lt	II	Insecticide	For the control of stalk	US EPA
Duduthrin	Lambda cylothrin (pyrethroid) 50 g/lt	II	Insecticide	For the control of stalk	US EPA
Duduba 450EC	Cypermethrin 10 g/lt + chloropyrifos 35 g/lt	II	Insecticide	For the control of tuber moth (PTM) and African boll worm (ABW)	US EPA
Duduall 450EC	Cypermethrin 150 g/lt + chloropyrifos 300 g/lt	II	Insecticide	For the control of tuber moth (PTM) and African boll worm (ABW)	US EPA

Key reference: US EPA: United State Environmental Protection Agency, (Preregistrations eligibility decision for triforine) [12,13,14].

Flower farms followed the procedures of pesticide management as per the direction of the manufacturer, but the interviewed pesticide applicators in onion farms revealed that 1% of stored pesticides that was easily accessible by children, 11% stored pesticide in closed containers, 54% stored pesticides in their farmhouse, 9% stored pesticides in the kitchen and 15% stored pesticides in the sleeping room.

### Use of personal protective equipment and hazardous practices among pesticide applicators

**Figure 1:**
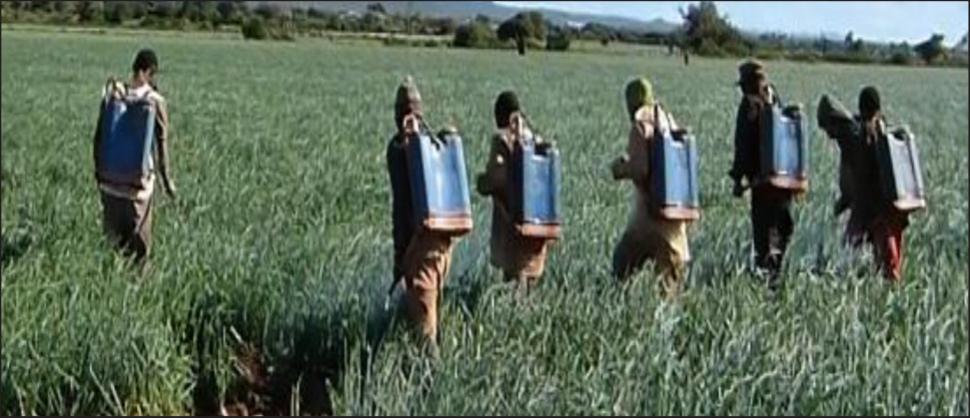
Pesticide applicators in an onion farm handling pesticides without PPE.

**Figure 2:**
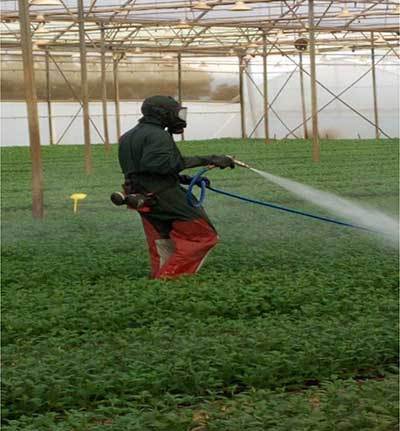
Applicator with PPE in a flower farm.

Among pesticide applicators in onion farms, 83.9% do spraying only and 16.1% carry out both spraying and pesticide preparation (mixing and loading). Wearing PPE is among the behaviours mostly assumed to protect workers from pesticide exposure. In terms of protective equipment, this study shows that no pesticide applicators in onion farms were observed to wear any protective measures like caps, gloves, full-sleeve shirts, face masks, and shoes during mixing, loading and spraying pesticides. They put on only shirts or T-shirts, shorts or trouser and slippers or no shoes at all (Figure 1). The situation was reversed in the flower farms, where all pesticide applicators used PPE that protected them from direct exposure to pesticides (Figure 2). The common PPE used were boots, gloves, full and a half face masks and coveralls.

**Figure 3:**
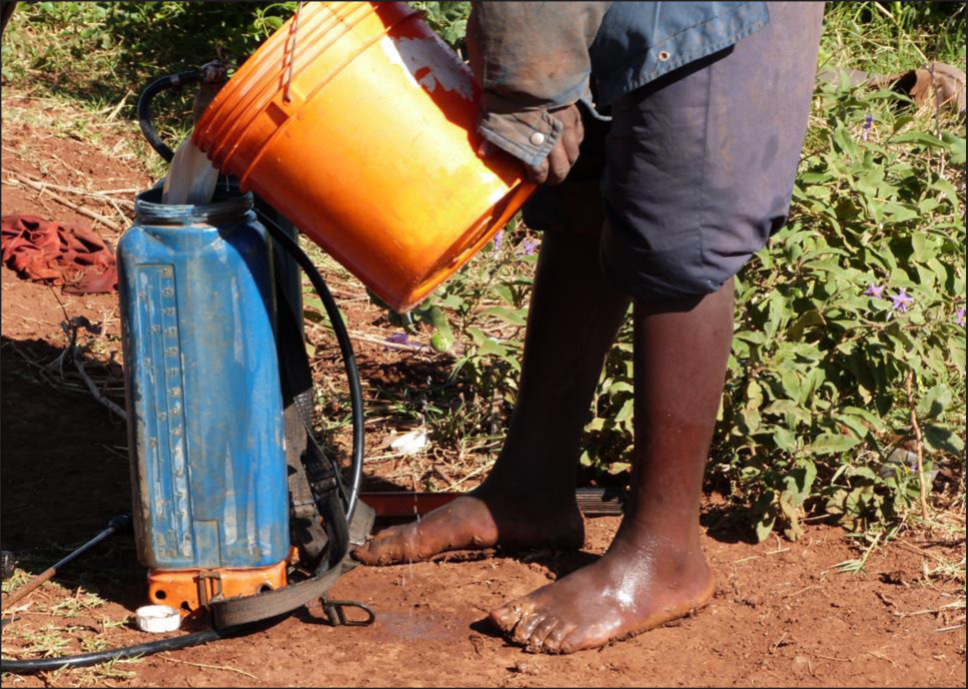
Pesticide spilled on body and cloth in onion farms.

**Figure 4:**
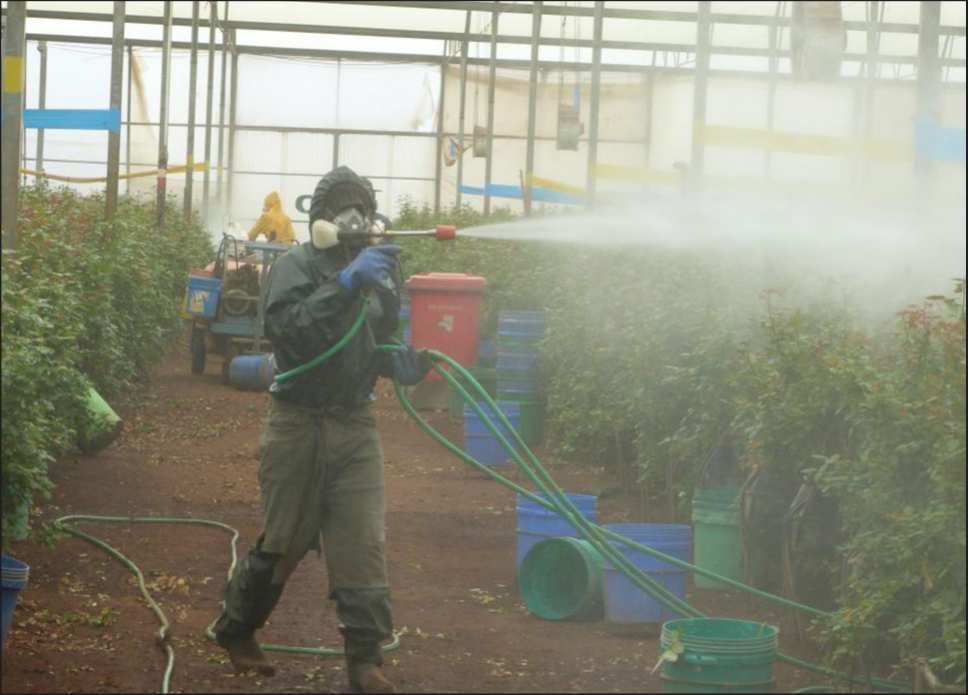
Pesticide spill on PPE at flower farm.

There were several hazardous practices identified with regard to pesticide handling. It was observed that 62.7% of pesticide applicators spilled pesticides on their body when they did spraying in flower and onion farms (Figures 3 and 4). Other hazardous practices in onion farms included: mixing pesticides with bare hands (40%) or using a stick (22%), applying pesticides with bare hands (40%) or with a hand-held sprayer (40%), not wearing protective equipment or clothing while handling pesticides (40%), not washing their hands before eating, smoking or using tobacco (5%), and not bathing or changing clothes after working with pesticides (12%).

**Figure 5:**
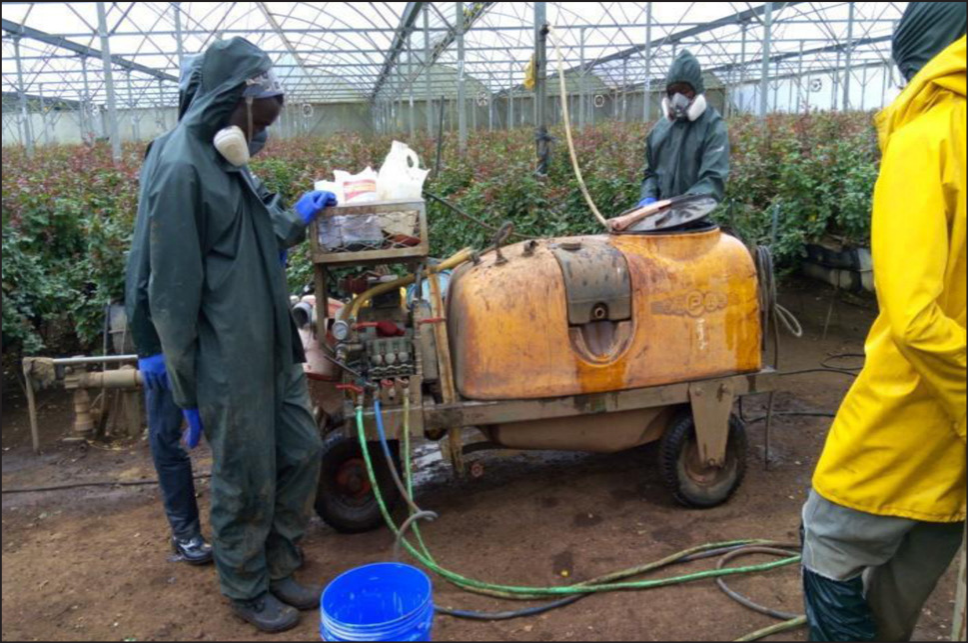
Motorized mobile pesticide sprayer in flower farm.

*The equipment used to spray pesticide.* Mobile motorized tanks with pipes and spray guns were used in flowers, whereby most of them had leakage and spilled pesticide on the ground that exposed applicators to the risk. Manual knapsack equipments were used in onions farms, which left the pesticide applicator to high exposure to pesticide because of leakages (Figure 5).

**Figure 6:**
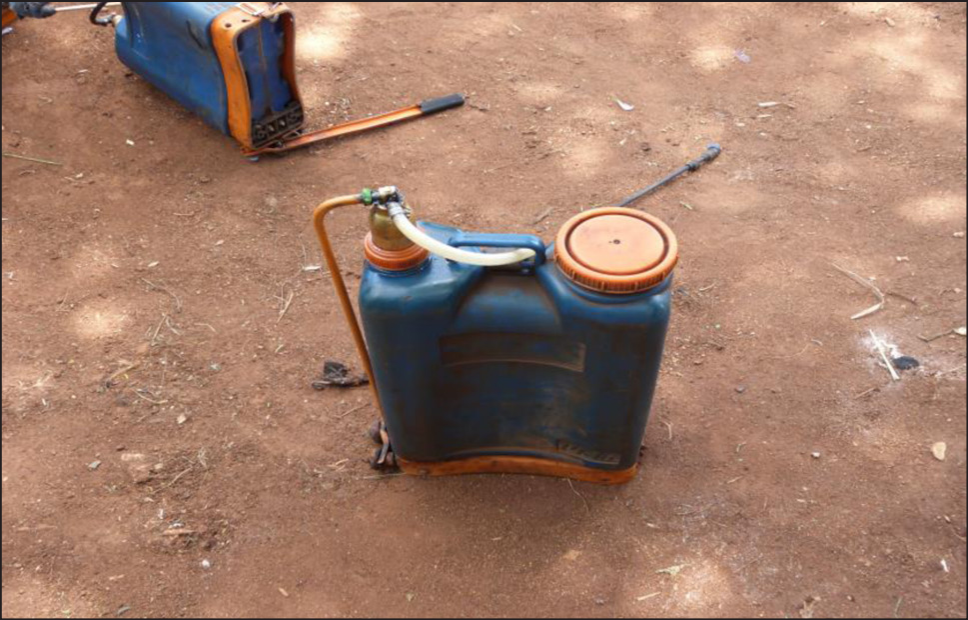
Manual knapsack equipment used in onion farms.

A manual knapsack was the equipment being used most frequently in onion farms for the spraying of pesticide on the field (Figure 6).

**Figure 7:**
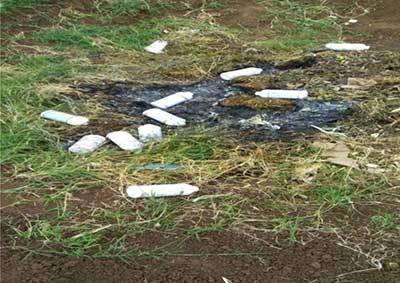
Empty pesticide containers disposed to the environment in the onion farm.

Empty pesticide containers were thrown in the field; river and home refuse collection pits in onion farms (Figure 7).

**Figure 8:**
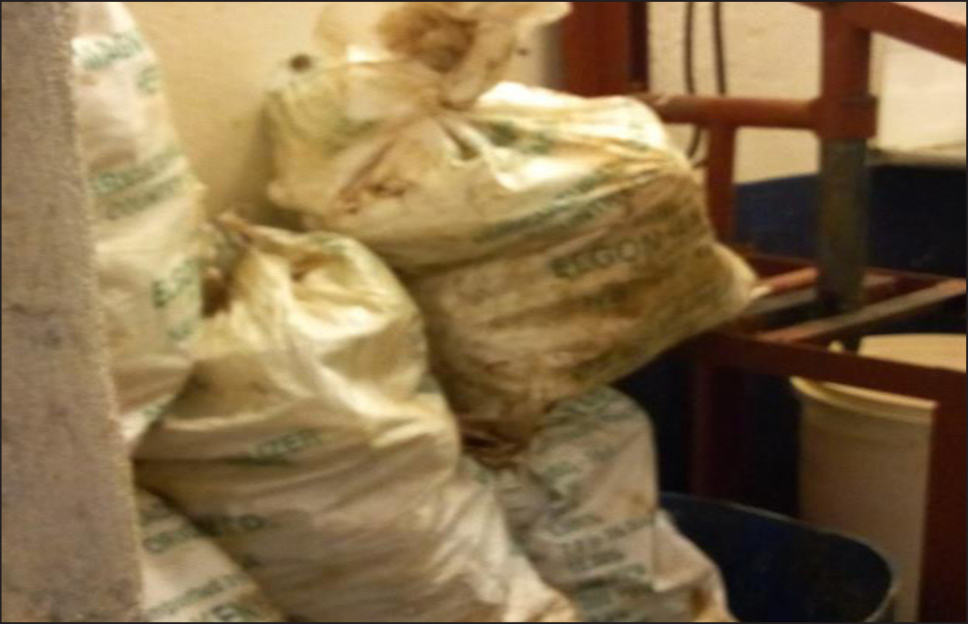
Empty pesticides stored in bags in flower farms.

However, in flower farms, empty pesticide containers were collected into plastic bags and then transported to the distraction unit identified by TPRI then transported for distraction (Figure 8).

### Neurological health symptoms experienced by pesticide applicators

Neurological symptoms were collected from all recruited study participants in both farms. The onion farmers reported more symptoms, with body weakness having the highest frequency (91.1%), followed by pain in part of the body (64.3%). The highest symptoms reported by flower farmers were excessive sweating (46.4%) followed by body weakness (34.5%) (Table 5).

**Table 5:** Proportion of neurological health symptoms among study participants in flower (n = 84) and onion farms (n = 56).

Neurological health symptoms	Flower farms (%)	Onion farms (%)

Excessive sweating	46.4	32.1
Body weakness	34.5	91.1
Abnormal tiredness of the body	28.6	55.4
Headache	27.4	58.9
Pain in part of the body	21.4	64.3
Poor appetite	17.9	46.4
Depression	15.5	17.9
Irritation	13.1	46.4
Nervousness or shakiness inside	11.9	23.2
Nausea	8.3	39.3
Dizziness	7.1	53.6
Ringing in ears	7.1	30.4
Trouble falling asleep	7.1	14.3
Feeling hot or cold	7.1	23.2
Vomiting	3.6	28.6
Loss of concentration	4.8	17.9

## Acetylcholinesterase Levels

### Reported neurological health symptoms in onion farms

About 39% of onion farmers and 19% of flower farmers had acetylcholinesterase levels below the normal range, whereas 60.7% from onion farms and 81% from flower farms had cholinesterase level above the limit level. The mean cholinesterase level among onion and flower farmers was 27.882 ± 3.829 U/g Hgb and 25.146 ± 3.9607 U/g Hgb, respectively. The mean average values of the AChE levels in flower and onion farm workers was 26.788 ± 4.0952 U/g Hgb with a paired T test of 1.301 and p < 0.195, which was not a statistically significant difference between the two groups. The relationship between AChE and irritation was significant in onion farms. For instance, among the pesticide applicators in onion farms who reported irritation, 20 (58.8%) had AChE levels within the normal range while 6 (27.3%) had AChE levels below the normal range (p < 0.05) (Table 6).

**Table 6:** Reported neurological health symptoms among pesticide applicators in relation to cholinesterase test (N = 56) in onion farms.

Neurological health symptoms	AChE below normal range (N = 22)	AChE within normal range (N = 34)	Total (N = 56)	Chi sq	p-value

	n (%)	n (%)	n (%)		

Abnormal tiredness	13(59.1)	18(52.9)	31(55.4)	0.204	0.785
Painful part of the body	13(59.1)	23(67.6)	36(64.3)	0.426	0.575
Irritation	6(27.3)	20(58.8)	26(46.4)	5.346	0.029*
Depression	11(50.0)	17(50.0)	28(50.0)	0.000	1.000
Loss of concentration	5(22.7)	5(14.7)	10(17.9)	0.586	0.448
Perspiration	4(18.2)	14(41.2)	18(32.1)	3.238	0.087
Headache	15(68.2)	18(52.9)	33(58.9)	1.282	0.282
Dizziness	12(54.5)	18(52.9)	30(53.6)	0.014	1.000
Heart or chest pain	4(18.2)	6(17.6)	10(17.9)	0.003	1.000
Poor appetite	10(45.5)	16(47.1)	26(46.4)	0.014	1.000
Vomiting	9(40.9)	7(20.6)	16(28.6)	2.703	0.134
Ringing in ears	6(27.3)	11(32.4)	17(30.4)	0.163	0.772
Nausea	10(45.5)	12(35.3)	22(39.3)	0.578	0.577
Trouble falling asleep	6(27.3)	6(17.6)	12(21.4)	0.735	0.508
Feeling hot or cold	7(31.8)	6(17.6)	13(23.2)	1.505	0.332
Body weakness	21(95.5)	30(88.2)	51(91.1)	0.856	0.340

Key:* Level of statistical significance (p value < 0.05).

Of the 24 pesticide applicators in flower farms who reported feeling abnormally tired, 20 (83.3%) had AChE levels below the normal range and 4 (28.6%) were within the normal range. Other neurological health symptoms (among the flower pesticide applicators with AChE levels below the normal range) that occur in high proportion include excessive sweating (45.6%), body weakness (36.8%) and headache (26.5%). For all reported symptoms, those with AChE levels below the normal range did not differ significantly from those with AChE level within the normal range (Table 7).

**Table 7:** Reported neurological health symptoms among pesticide applicators in relation to cholinesterase level (N = 84) in flower farms.

Neurological health symptoms	AChE below normal range	AChE within normal range	Total	Chi sq	p-value

	n (%)	n (%)	n (%)		

Abnormal tiredness	20(83.3)	4(16.7)	24(28.6)	0.124	1.000
Painful part of the body	14(20.6)	4(25.0)	18(21.4)	0.150	0.739
Irritation	9(13.2)	2(12.55)	11(13.1)	0.060	1.000
Depression	9(13.2)	4(25.0)	11(15.5)	1.370	0.260
Loss of concentration	4(5.9)	0(0.0)	4(4.8)	0.988	1.000
Excessive sweating	31(45.6)	8(50.0)	39(46.4)	0.101	0.787
Headache	18(26.5)	5(31.2)	23(27.4)	0.149	0.758
Dizziness	5(7.4)	1(6.2)	6(7.1)	0.024	1.000
Heart or chest pain	7(10.3)	1(6.2)	8(9.5)	0.246	1.000
Poor appetite	13(19.1)	2(12.5)	15(17.9)	0.387	0.725
Vomiting	3(4.4)	0(0.0)	3(3.6)	0.732	1.000
Ringing in ears	6(8.8)	0(0.0)	6(7.1)	1.520	0.590
Nausea	7(10)	0(0)	7(8.3)	0.246	1.000
Trouble falling asleep	7(10)	1(6.2)	8(9.5)	0.246	1.000
Feeling hot or cold	6(8.8)	0(0.0)	6(7.1)	1.52	0.590
Body weakness	25(36.8)	4(25.0)	29(34.5)	0.793	0.560

## Discussion

This study demonstrates a significantly high proportion of neurological health symptoms in onion pesticide applicators when compared to pesticide applicators in flower farms, with a greater risk of pesticide exposure. This was due to lack of personal protective equipment for pesticide applicators in onion farms compared to those of flower farms, which poses a greater risk of cholinesterase inhibition and neurological health symptoms to pesticide applicators. The results of this study can be compared with the results of other, similar studies. This study aimed to assess neurological health symptoms associated with pesticide exposure among flower and onion pesticide applicators.

The study results show that more 98% of pesticides used in the study area were organophosphates and a small amount was carbamate. These results were similar to the study done in Gazoué and Savè townships, in the central republic of Benin on risk factors of pesticide poisoning and pesticide users’ cholinesterase levels among farmers, which found that 72.96% of pesticide used were organophosphates and pyrethroids constituted the group of pesticides more frequently used by 88% of the farmers [15]. In addition, it is necessary to note that among the problems mentioned by pesticide applicators was a lack of knowledge about the handling of pesticides. Their presence reveals the existence of sources for the informal provision of pesticides, opening the door to increased risks of poisoning. The predominance of the organophosphates in this study showed the importance of monitoring cholinesterase activity as the main test of poisoning with pesticides.

The study provides insights into pesticide application and related health symptoms among flower and onion pesticide applicators in Arusha. A majority of pesticide applicators (75%) in the studied region suffered from one or more forms of pesticide-related health symptoms. This result was supported by the findings of the study done in Nigeria on pesticide use practices and safety issues, which reported that about 80% of both farm workers indicated that they experience discomforts such as headaches, tiredness, vomiting, nausea and skin problems (itching and skin burn) after spraying [16].

A group of pesticide applicators from onion farms with high level of exposure to pesticide was found to have higher prevalence of body weakness (91.1%), headache (58.9%), dizziness (53.6%), irritation (46.4%) and feeling cold or hot (23.2%), whereas flower farms showed the a lower prevalence of body weakness (91.1%), headache (27.4%), dizziness (7.1%), irritation (13.1%) and feeling cold or hot (7.1%) for pesticide applicators. These findings were similar to the study done in Zimbabwe at Kwekwe district on health effects of agrochemicals, where headache (66.7%), cold/flu (62.2%), weakness (45.9%), dizziness (41.1%) and skin irritation (39.0%) were reported among farm workers in commercial farms [17].

The statistically significant depletion in AChE activity clearly exhibits the exposure to organophosphate (OP) and carbamate pesticides among pesticide applicators. In the present study, the mean average of AChE level among pesticide applicators in onion farms was greater by 2.736 U/g Hgb as compared to those of flower farms, which had a mean concentration of 25.146 ± 3.9607 U/g Hgb. The findings were in agreement with a study done in Tanzania on acute health effects of organophosphorus pesticides on Tanzanian, whereby the mean was 32.0 U/g Hgb [11].

Studies have shown that the blood AChE level must be depressed to less than 20% of its normal value before symptoms of systemic poisoning appear [18]. In the present study, there was no indication of such depression, which explains the relationship between symptoms and AChE, even if the proportion of the participants with depressed numbers was observed to be higher than those who were above the limit.

The failure of farmers to use PPE during pesticide application presents potential risks to pesticide exposure. The results indicated that 100% of the onion farms’ pesticide applicators in the study used no form of PPE during pesticide application; however, in flower farms the reverse was true: pesticide applicators put on full PPE during pesticide application. Forty percent of farmers apply pesticides with no PPE, while a majority of farmers (60%) put on PPE during pesticide application, of the total pesticide applicators involved in the study. Wearing or putting on full PPE during pesticide application in this study was defined as wearing a nose mask, hand rubber glove, overall, long coat, facemask and boot (rubber boot) at the time of application. Alternatively, applying pesticides without PPE connotes when a farmer uses casual farming clothes without any of the listed PPEs. The findings in this study are in line with a study done on Cambodian farmers that shows a reduction of risk of acute pesticide poisoning by 55% among more highly educated farmers who adopted extra personal protective measures [18]. The study on community-based intervention to reduce pesticide exposure to farm workers and potential take-home exposure to their families showed the importance of wearing protective gloves in reducing pesticide exposures among strawberry harvesters [19]. The study done in Ghana on farmer perceptions and pesticide use practices in vegetable production showed that pesticide poisoning occurred more often among farmers who generally did not wear protective clothing during spraying of pesticides [20]. In addition, a study of farmers who used pesticides in rural Indonesia, showed that those who wore no mask/respirator, wet clothing or short sleeves had greater skin contact with pesticides [21].

Based on sufficient evidence from this study, pesticide applicators were exposed to pesticide above the acceptable level. Those who did not use PPE were more likely to develop neurological symptoms compared to those who used PPE. Therefore, the use of PPE is unavoidable and should always be encouraged during application of pesticides.

## Conclusion

Our study shows that a significant percentage of respondents from flower and onion farms are suffering from neurological symptoms that could be related to pesticide exposure. Those working in the agricultural sector requiring direct contact with pesticides but with less training for the majority of pesticide applicators, low knowledge of and lack of PPE use and high duration of exposure among pesticide applicators are more at risk from accidental pesticide poisoning, based on self-reporting (determined via questionnaire). No one, therefore, is exempted from the adverse effects of pesticides as long as these chemicals are readily available and being used in flower and onion farms in the given area of study.

## Recommendation

Pesticide applicators should receive training concerning pesticide management on various onion farms as is provided to flower pesticide applicators, where pesticide applicators were aware about the risks of pesticide when being exposed to it. Also, introduction of integrated pest management practices wherever possible to reduce exposures and adverse effects arising from application of pesticides, a practice that has lost favor because of safety concerns, was important. In addition, further research is needed to ascertain causality regarding such high frequency of neurological symptoms.
